# Neuropsychiatric symptoms of dementia and caregiver burden: a systematic review and meta-regression

**DOI:** 10.1590/1980-5764-DN-2025-0369

**Published:** 2026-07-24

**Authors:** Carolina Braga Moura, Ana Maria Porto, Luisa Lara Calazans, Leonardo Sena, Marianna Leite, Fernando Rodríguez González, Matheus Nunes Ferreirinha Leite de Castro, Raquel Quimas Molina da Costa

**Affiliations:** 1 Universidade Federal Fluminense, Hospital Universitário Antônio Pedro, Ebserh, Niterói RJ, Brazil.; 2 Universidade Federal Fluminense, Faculdade de Medicina, Niterói RJ, Brazil.; 3 Faculdade de Medicina Santa Marcelina, São Paulo SP, Brazil.; 4 Universidade Federal Fluminense, Faculdade de Medicina, Programa de Pós-Graduação em Neurologia e Neurociências, Niterói RJ, Brazil.

**Keywords:** Dementia, Behavioral Symptoms, Caregivers, Caregiver Burden, Demência, Sintomas Comportamentais, Cuidadores, Sobrecarga do Cuidador

## Abstract

Managing neuropsychiatric symptoms is crucial in dementia. The Neuropsychiatric Inventory-Distress (NPI-D) assesses caregiver distress, and the Zarit Burden Interview (ZBI) evaluates overall burden. Objective: This meta-regression examined the relationship between ZBI and NPI-D with NPI total scores to explore how burden relates to caregiver distress and neuropsychiatric symptoms. Methods: After a systematic review of studies addressing neuropsychiatric symptoms and caregivers’ burden in people with dementia, a meta-regression was conducted to examine the association between NPI-D and ZBI scores and NPI total scores. We used data from five studies reporting NPI-D scores and seven reporting ZBI scores. Single-arm meta-analyses for NPI-D, NPI total, and ZBI scores were performed using a random-effects model. Results: The random-effects model estimated a mean ZBI score of 23.76 [95% confidence interval - 95%CI 14.32-33.20, I2=100%, p=0]. NPI-D scores had a mean of 10.09 [95%CI 6.56-13.63, I2=96%, p<0.01], and the mean NPI total score was 21.84 [95%CI 19.31-24.38, I2=95%, p<0.01]. The meta-regression between NPI-D Mean and NPI total scores showed a statistically significant relationship, with a coefficient of 1.32 [95%CI 0.97-1.68, p=0.000], indicating a strong association between caregiver distress and neuropsychiatric symptoms. Conclusion: Single-arm studies suggest a positive relationship between neuropsychiatric symptoms and caregiver burden. The meta-regression analysis between NPI-D and NPI total demonstrates a significant link between caregiver distress and neuropsychiatric symptoms. Future studies should develop precise tools capturing caregivers’ challenges and burden.

**PROSPERO:**

CRD42024567953

## RESUMO.

O manejo dos sintomas neuropsiquiátricos é fundamental na demência. O Neuropsychiatric Inventory-Distress (NPI-D) avalia a angústia do cuidador, enquanto o Zarit Burden Interview (ZBI) mede a sobrecarga global. Objetivo: Esta metarregressão examinou a relação entre os escores do ZBI e NPI-D e os escores totais do NPI, a fim de explorar como a sobrecarga se relaciona à angústia do cuidador e aos sintomas neuropsiquiátricos. Métodos: Após uma revisão sistemática de estudos que abordaram sintomas neuropsiquiátricos e sobrecarga de cuidadores em pessoas com demência, realizou-se uma metarregressão para avaliar a associação entre os escores do NPI-D, do ZBI e os escores totais do NPI. Foram utilizados dados de cinco estudos que reportaram escores de NPI-D e sete que reportaram escores de ZBI. Metanálises single-arm para os escores do NPI-D, NPI total e ZBI foram conduzidas utilizando um modelo de efeitos aleatórios. Resultados: O modelo de efeitos aleatórios estimou um escore médio do ZBI de 23,76 [intervalo de confiança de 95% - IC95% 14,32-33,20; I2=100%; p=0]. Os escores do NPI-D apresentaram média de 10,09 [IC95% 6,56-13,63; I2=96%; p<0,01], e o escore médio do NPI total foi de 21,84 [IC95% 19,31-24,38; I2=95%; p<0,01]. A metarregressão entre a média do NPI-D e os escores totais do NPI mostrou associação estatisticamente significativa, com coeficiente de 1,32 [IC95% 0,97-1,68; p=0,000], indicando forte relação entre a angústia do cuidador e os sintomas neuropsiquiátricos. Conclusão: Estudos single-arm sugerem relação positiva entre sintomas neuropsiquiátricos e sobrecarga do cuidador. A análise de metarregressão entre NPI-D e NPI total demonstra associação significativa entre a angústia do cuidador e os sintomas neuropsiquiátricos. Pesquisas futuras devem desenvolver instrumentos mais precisos para captar os desafios e a sobrecarga enfrentados pelos cuidadores.

## INTRODUCTION

Dementia is a significant and growing public health challenge, which can severely impair patients’ functionality and result in a high degree of dependence on caregivers^1^. The burden experienced by caregivers has emerged as a critical area of research, due to its profound implications for both parties’ well-being^2^. Caregiver burden imposes physical, psychological, social and financial strains^2^ ^,^ ^3^ ^,^ ^4^. Caregivers of patients living with dementia are more likely to experience a higher level of overload, compared to other chronic illness patients, and carry a higher risk of cardiovascular diseases, anxiety, depression and sleep disorders^5^ ^,^ ^6^. Several validated scales, including the Zarit Burden Interview (ZBI) and the Neuropsychiatric Inventory Caregiver Distress Scale (NPI-D), are used to assess this burden. The impact is particularly observed on family members, often women, who experience higher burden levels than men^5^ ^,^ ^7^ ^,^ ^8^.

Behavioral and Psychological Symptoms in Dementia (BPSD) can significantly contribute to caregiver burden, which is influenced by the stage of the disease, increasing stress, guilt, and distress^9^ ^,^ ^10^ ^,^ ^11^. A tool commonly used for the assessment of BPSD is the Neuropsychiatric Inventory (NPI), which evaluates ten behavioral and two neurovegetative manifestations^12^. Symptoms such as apathy, depression and delusions are particularly difficult to manage, thus being a frequent reason for institutionalization and escalating care costs^8^ ^,^ ^11^ ^,^ ^13^. Effective management of BPSD may alleviate caregiver burden and, despite the growing body of literature analyzing this association, few studies have focused on the impact of each specific symptom and the differences between the prevalence and impact of BPSD^14^.

Our aim was to identify which neuropsychiatric symptoms contribute the most to caregiver burden in people living with dementia, allowing future interventions to address them. By pinpointing and managing these symptoms more effectively, we can alleviate caregivers’ burden, ultimately improving their well-being. Reducing caregiver burden not only enhances quality of life for those providing care but also promotes better patient outcomes. Through this approach, we hope to offer clear, evidence-based guidance that supports the decision-making process of healthcare providers and caregivers, easing the multifaceted challenges they face.

## METHODS

### Eligibility criteria

Inclusion in this meta-analysis was restricted to studies that met all the following eligibility criteria:

- randomized controlled trials, cross-sectional cohorts or retrospective cohorts;
- caregiver burden assessment with NPI-D or ZBI;
- inclusion of BPSD prevalence data;
- patients diagnosed with any etiology of dementia.

We excluded studies with:

- no detailed BPSD symptoms prevalence; and
- no objective measure of caregiver burden.

### Search strategy and data extraction

We systematically searched United States National Library of Medicine (PubMed), Scientific Electronic Library Online (SciELO), Embase, and Latin American and Caribbean Health Sciences Literature (LILACS). Central Register of Controlled Trials from inception to September 2024 with the following search terms: “Caregiver burden AND dementia AND NPI” for PubMed, “Caregiver burden AND dementia AND neuropsychiatric inventory questionnaire” in Embase, “Exaustão do Cuidador AND demência” in SciELO and “Exaustão do Cuidador” AND Demência” in LILACS. The references from all included studies, previous systematic reviews and meta-analyses were also searched manually for any additional studies. Three authors (A.M, L.S. and L.C) independently extracted the data following predefined search criteria and quality assessment.

### Endpoints and subanalyses

Clinical outcomes included BPSD symptom frequency and caregiver burden (NPI-D and ZBI scores). Caregiver burden scales were reported in Table 1. We also reported characteristics (e.g., age, patients, dementia severity) and methodological variables (e.g., sample size, study quality). All analyses were conducted using the R software, with statistical significance set at p<0.05.

**Table 1.** Baseline characteristics of included studies.

Study	Study design	Patients	CG	Female - CG (%)	MMSE	Age - Patients	Age - CG	NPI total	NPI-D score	ZBI score
Baharudin et al.^17^	Cross-sectional	202	172	114 (66.3)	-	-	-	-	-	-
Dauphinot et al.^6^	Cross-sectional	548	548	-	18.98±6.39	81.10±7.21	-	21.03±17.17	-	3.6±2.0
García-Martin et al.^7^	Cross-sectional	129	125	97 (77.6)	-	-	60.7±14.3	24.9±3,4	12.5±1.4	12.3±0.7
Hanzevachi et al.^18^	Cross-sectional	131	131	89 (68)	15	80	60	26	-	27
Huang et al.^15^	Cross-sectional	88	88	46 (52.3)	13.8±6.5	80.0±7.7	54.6±14.1	20.3±21.0	8.8±10.9	-
Kamalzadeh et al.^3^	Cross-sectional	60	60	45 (75)	15.74±6.85	74.05±9.32	47.22±12.20	24.55±19.09	-	46.11±21.62
Matsumoto et al.^16^	Cross-sectional	67	67	52 (77.7)	20.1±5.2	80.8±7.0	63.58±10.9	13.38±13.9	4.68±5.6	19.68±14.8
Melo et al.^8^	Cross-sectional	105	105	72 (68.6)	13.9±7.9	75.4±8.1	67.0±12.5	26.4±17.1	15.2±10.3	31.8±14.3
Regier et al.^13^	RCT	250	250	203 (81.2)	14.3±7.8	81.4±7.9	65.4±12.6	-	-	-
Tan et al.^10^	Cross-sectional	85	-	-	10.07±7.98* 10.74±5.90^†^	76.14±7.36	-	28.79±17.63* 27.07±19.74^†^	15.95±8.11^‡^ 9.89±6.52^§^	-
Thasleem et al.^4^	Cross-sectional	71	-	70 (98.6)	-	80.03±7.67	50.38±13.21	-	-	-
Thyrian et al.^19^	PC	171	171	126 (73.7)	20.87±5.6	79.79±5.4	64.51±13.72	11.91±16.0	-	-
Truzzi et al.^9^	Cross-sectional	159	159	131 (82.4)	14.9±6.8	76.9±6.9	56.2±13.3	21.4±18.6	9.4±8.5	-
Tsai et al.^5^	PC	509	509	0	15.39±7.01	77.83±7.98	56.76±14.08	-	-	26.9±15.8
Zwijsen et al.^11^	Cross-sectional	432	-	418 (96.8)	-	83.3±7.6	41±12	-	-	-

Abbreviations: CG, caregiver ; MMSE, Mini-Mental State Examination; NPI, Neuropsychiatric Inventory; NPI-D, Neuropsychiatric Inventory-Distress; ZBI, Zarit Burden Interview; RCT, randomized controlled trial; PC, Prospective Cohort.

Notes: *Inpatient; †Outpatient; ‡Family carer; §Professional carer.

The study design specifies whether the study is transversal (cross-sectional), a prospective cohort, or a randomized controlled trial. Patients indicate the number of dementia patients included in each study, while CG represents the number of caregivers assessed. The Mini-Mental State Examination was used as a cognitive test, ranging from 0 to 30. The Age - Patients, and Age - CG columns provide the average age for patients and caregivers, respectively. The Neuropsychiatric Inventory evaluates neuropsychiatric symptoms in dementia patients, and average total scores are reported in the column Neuropsychiatric Inventory total. The Neuropsychiatric Inventory-Distress Score (NPI-D score) quantifies the level of distress experienced by caregivers due to the patient's neuropsychiatric symptoms. Caregiver burden is assessed using the Zarit Burden Interview (ZBI score), a questionnaire in which higher scores indicate a greater perceived burden. The Female - CG column reports the number and percentage of female caregivers in each study. Average values are presented as Mean and Standard Deviation or Median and Interquartile range. The randomized controlled trial design is highlighted in studies that involve intervention comparisons with random participant assignment.

Source: The authors.

### Statistical analysis

This systematic review and meta-analysis was performed in accordance with the Cochrane Collaboration and the Preferred Reporting Items for Systematic Reviews and Meta-Analysis (PRISMA) statement guidelines^20^. Odds-ratios (OR) with 95% confidence intervals were used to calculate the proportion of burden scores and BPSD. Cochran Q test and I2 statistics were used to assess for heterogeneity; P values inferior to 0.10 and I2>25% were considered significant for heterogeneity. Single-arm meta-analyses for NPI-D, NPI total, and ZBI scores were performed using DerSimonian and Laird random-effects. In addition, a random-effect meta-regression analysis was performed to examine the association between NPI-D scores and NPI total, as well as individual symptom scores. Meta-regressions were conducted using data from five studies reporting NPI-D scores and seven reporting ZBI scores. R software (version 2024.04.2+764) was used for statistical analysis.

## RESULTS

### Study selection and characteristics

The initial search yielded 341 results. After the removal of 97 duplicate records and 201 ineligible studies, 43 remained and were fully reviewed based on the inclusion criteria. Of these, a total of 15 studies were included, comprising 3,007 patients from 14 non-randomized cohorts and one randomized controlled trial (RCT). A total of 2835 caregivers were interviewed.

### Frequency of neuropsychiatric symptoms

**Figure 1.**
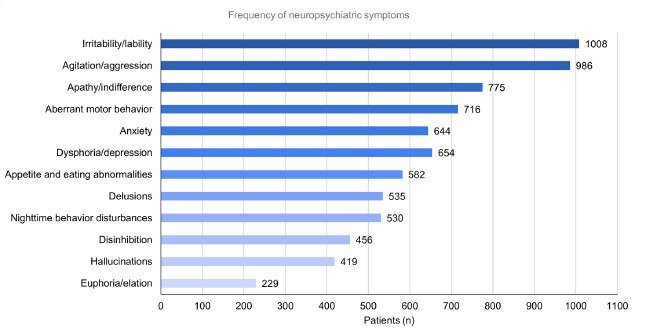
Frequency of neuropsychiatric symptoms.

Notes: Total number of patients (n) presenting each neuropsychiatric symptom assessed in the Neuropsychiatric Inventory.

Source: The authors.

The frequency of neuropsychiatric symptoms among patients varied considerably across different symptom categories (Figure 1). Irritability/lability was the most prevalent symptom, affecting 1,008 patients, followed closely by agitation/aggression, which was reported in 986 cases. Apathy/indifference was also frequent, observed in 775 patients. Aberrant motor behavior was identified in 716 individuals, while anxiety and dysphoria/depression were present in 644 and 654 patients, respectively. Appetite and eating abnormalities were reported in 582 patients, and delusions in 535 cases. Nighttime behavior disturbances were documented in 530 individuals. Less common symptoms included disinhibition (456 patients), hallucinations (419 patients), and euphoria/elation, which was the least frequently reported, affecting 229 patients. Individual RCT appraisal is reported in Table 1.

### Neuropsychiatric Inventory-Distress and Zarit Burden Interview average mean scores by neuropsychiatric symptoms

The Neuropsychiatric Inventory-Distress (NPI-D) scores varied across different symptoms, with agitation (1.88; 95%CI 0.89-2.87), delusions (1.85; 95%CI 0.74-2.96), and irritability (1.73; 95%CI 0.71-2.75) being the most burdensome for caregivers. Apathy (1.68; 95%CI 0.85-2.52) and anxiety (1.47; 95%CI 0.56-2.37) also had high distress scores. In contrast, euphoria (0.38; 95%CI 0.04-0.71) was associated with the lowest burden.

For ZBI scores, the highest burden was reported for nighttime disturbances (46.50±18.72), followed by euphoria (43.54; 95%CI 4.24-82.83) and hallucinations (42.13 95%CI 14.32-69.94). Similarly, disinhibition (42.13; 95%CI 9.59-74.66) and anxiety (38.91; 95%CI 13.85-63.96) were among the most burdensome symptoms. The lowest ZBI burden was associated with apathy (26.32; 95%CI 2.68-49.96) and irritability (26.75; 95%CI 0.83-52.68), while appetite changes (27.05; 95%CI -17.98-72.08) had a wider range of reported distress.

### Pooled analysis of studies

**Figure 2.**
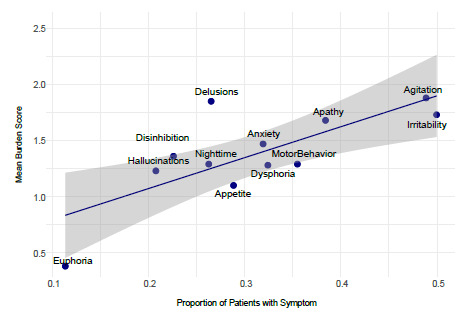
Meta-regression of the relationship between the prevalence of each neuropsychiatric symptom and caregiver distress measured by the Neuropsychiatric Inventory-Distress.

Notes: This figure shows the results of a meta-regression assessing the correlation between the proportion of patients presenting each neuropsychiatric symptom in the Neuropsychiatric Inventory and the mean total scores of caregiver’s distress, as measured by the NPI-D. The regression coefficient was 1.324 (95%CI 0.97-1.678; p<0.001).

Source: The authors.

**Figure 3.**
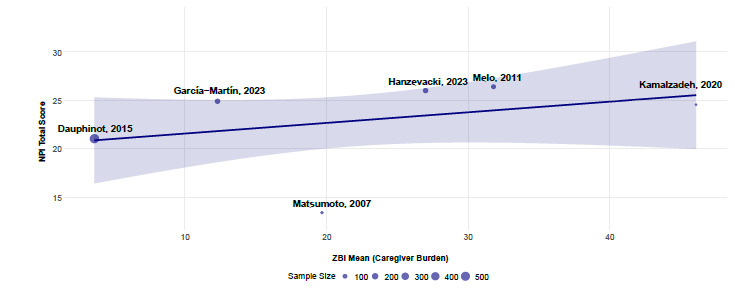
Meta-regression of the relationship between Neuropsychiatric Inventory total score and caregiver burden measured by Zarit Burden Interview.

Notes: Results of the meta-regression assessing the correlation between the mean total scores from the Neuropsychiatric Inventory and the mean total score from the Zarit Burden Interview, in each study. The regression coefficient was 0.11 (95%CI -0.09-0.31; p=0.2818). The sample size from each study is demonstrated by the size of the blue dots.

Source: The authors.

**Figure 4.**
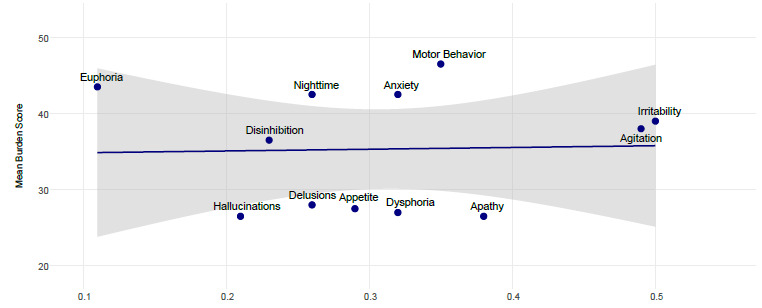
Meta-regression of symptom prevalence measured by the Neuropsychiatric Inventory and caregiver burden measured by the Zarit Burden Interview.

Notes: Relationship between the proportion of patients presenting each neuropsychiatric symptom measured by the Neuropsychiatric Inventory and mean scores of caregiver burden, measured by the Zarit Burden Interview. A modest positive association was observed, which was not statistically significant (regression coefficient 0.034; 95%CI -0.551-0.596; p=0.9169). The shaded region around the line indicates the confidence interval for this trend. A wider region implies less statistical certainty.

Source: The authors.

The random-effects model estimated a mean ZBI score of 23.76 [95%CI 14.32-33.20, I^2^=100%, p=0]. NPI-D scores had a mean of 10.09 [95%CI 6.56-13.63, I^2^=96%, p<0.01], and the mean NPI total score was 21.84 [95%CI 19.31-24.38, I^2^=95%, p<0.01]. The meta-regression between NPI-D Mean and NPI total showed a statistically significant relationship, with a coefficient of 0.755 [95%CI 0.319-0.927, p=0.004], indicating a strong association between caregiver distress and neuropsychiatric symptoms using those scales. Other results from the meta-regression analysis are shown in Figures 2 to 4. It is important to note that the pooled analysis presented in Figures 3 and 4 includes only a subset of studies. This selection was based on the availability of both ZBI and NPI data, as only studies reporting these two outcomes were eligible for this specific analysis. Consequently, studies that did not provide both measures were excluded from this particular pooled estimation to ensure comparability and methodological consistency.

## DISCUSSION

This systematic review and meta-regression showed a statistically significant association between caregiver distress and neuropsychiatric symptoms in people living with dementia, as suggested by previous single-arm studies^6^ ^,^ ^9^ ^,^ ^11^. What distinguishes this review from previous literature is the evaluation of symptom prevalence and the impact on caregiver burden for each individual neuropsychiatric symptom across a broad spectrum of dementia diagnoses. By encompassing all caregiver-patient dyads regardless of dementia etiology, this approach provides a more nuanced understanding of which symptoms are not only frequent but also disproportionately distressing to caregivers. Furthermore, meta-regression analysis between NPI-D and total NPI demonstrated how each neuropsychiatric symptom correlates proportionally to the amount of burden, as certain symptoms create unique challenges that affect caregivers in distinct ways^21^ ^,^ ^22^. Major BPSD includes agitation, depression, anxiety, apathy, delusions, hallucinations, and disrupted sleep patterns^7^ ^,^ ^8^ ^,^ ^9^ ^,^ ^15^ ^,^ ^16^.

In this study, we found that irritability/lability, ­agitation/aggression, apathy/indifference, and aberrant motor behavior were the most prevalent symptoms, as observed in previous studies, possibly due to the progressive neurological deterioration characteristic of the disease and emotional dysregulation^3^ ^,^ ^4^ ^,^ ^6^ ^,^ ^7^ ^,^ ^8^ ^,^ ^9^ ^,^ ^10^ ^,^ ^11^ ^,^ ^13^ ^,^ ^20^. Apathy, often characterized by a lack of motivation and emotional engagement, can exacerbate patients’ functional dependence, as they become reliant on caregivers to initiate and carry out daily activities despite retaining the physical capacity to do so^22^. This places additional strain on caregivers, fostering frustration and emotional detachment, which in turn weakens the caregiver-patient relationship and elevates emotional distress. Agitation, including physically or verbally aggressive behaviors, demands increased physical and emotional effort from caregivers^23^. These behaviors often precipitate feelings of embarrassment, frustration, or guilt, depending on their nature, and are strongly associated with decisions to institutionalize patients. Lastly, irritability, marked by mood instability and hypersensitivity to stimuli, can lead to heightened tension within the caregiving dynamic^24^.

Results from the meta-regression analysis suggest that more frequent symptoms tend to cause higher burden on average. We found a positive association between the prevalence of neuropsychiatric symptoms and caregiver burden, especially in the study from Garcia-Martín et al.^7^, suggesting that, as the proportion of patients experiencing a symptom increases, the average burden reported for that symptom rises. Agitation is a prevalent symptom in people living with dementia; it was also one of the most prevalent in this meta-analysis, and it is significantly associated with an increase in caregiver burden. However, not all symptoms in our analysis followed this pattern, and in certain cases, the prevalence of symptoms does not necessarily correlate with greater daily distress for the caregiver.

Although anxiety and nighttime behavior were not the most frequent symptoms, they were significantly associated with the overload of caregivers, following agitation, irritability, and apathy. It is probably because those symptoms may cause more immediate distress and demand frequent management efforts from caregivers^25^ ^,^ ^26^. Patients with dementia often experience sleep disturbances, including waking up frequently during the night, confusion, and agitation. These disruptions can prevent caregivers from getting adequate rest, leading to fatigue, irritability, and decreased ability to provide effective care. The lack of sleep can exacerbate stress levels, negatively affecting the caregiver’s physical and mental health^27^. Anxiety in patients with dementia often manifests as restlessness, fear, or agitation, particularly during the evening^28^. Caregivers may struggle to comfort the patient, and the inability to alleviate the patient’s distress can lead to feelings of helplessness or frustration^29^. This emotional burden can be challenging, especially if the anxiety is persistent and difficult to manage.

Delusions, euphoria, and appetite were not statistically significantly associated with caregiver distress. We hypothesize that this may be due to the nature of these symptoms being less immediately disruptive to caregivers’ daily routines or emotional well-being compared to more distressing symptoms such as agitation, anxiety, or nighttime disturbances. For instance, delusions may not always be perceived as immediately distressing by caregivers, as they may overlook or misinterpret the thoughts and perceptions expressed by patients^30^. However, the caregiver’s attempts to challenge or dispute the patient’s version of reality can inadvertently escalate aggressive behaviors, which can be particularly stressful and lead to heightened caregiver distress. In contrast, symptoms like agitation or anxiety are more directly confrontational and challenging to manage, which may explain their stronger association with caregiver burden^8^ ^,^ ^9^.

Similarly, euphoria, while potentially difficult to manage, might not provoke the same level of distress. Appetite changes, although important for overall health and family expenses, might not be as directly linked to the daily caregiving burden compared to symptoms that require more constant attention and intervention.

Regarding the distress of the caregiver, emotional and mental strain, physical exhaustion, social isolation, financial strain, and overall quality of life may be affected and are quantified by the ZBI score^31^. In this study, the lack of a significant association between symptom prevalence (measured by NPI) and caregiver burden (measured by ZBI) may be attributed to several factors. One possible reason is the limited number of studies included in the meta-regression, which could have reduced statistical power and increased variability in the estimates. Additionally, caregiver burden is influenced by multiple factors beyond symptom prevalence, such as the duration of caregiving, availability of support systems, and individual coping mechanisms. Caregivers must navigate unpredictable outbursts or negative interactions, which further erode their emotional well-being and contribute to cumulative stress. In turn, this may result in decreased patient care, higher rates of neglect, and a negative impact on the patient’s overall health, further exacerbating the symptoms of dementia. Moreover, a caregiver’s emotional state, such as heightened frustration or irritability, can contribute to a tense environment, which may increase the patient’s anxiety or agitation, creating a vicious cycle.

Thus, treatment strategies should prioritize addressing not only the most prevalent symptoms but the ones that burden the caregiver the most as well. For apathy, non-pharmacological interventions, such as structured activities, music therapy, and caregiver training to foster patient engagement can reduce functional dependency and prevent emotional detachment. For agitation, behavioral approaches to identify and manage triggers, environmental modifications, and, when necessary, judicious use of atypical antipsychotics or antidepressants may be indicated in some cases.

Managing irritability and anxiety involves creating a calm and predictable environment, employing relaxation techniques, and considering selective serotonin reuptake inhibitors (SSRIs) for mood stabilization when severe. Treating sleep problems in dementia involves a combination of strategies, including establishing a consistent sleep routine, improving the sleep environment, addressing underlying medical conditions, and managing nighttime agitation. Behavioral interventions like bright light therapy can help regulate the sleep-wake cycle, while physical activity and a balanced diet support better sleep. Reducing caffeine and alcohol intake, limiting daytime napping, and providing psychosocial support for caregivers are also important. In some cases, medications such as melatonin or sedative-hypnotics may be used cautiously. The approach should be individualized and include both patient and caregiver well-being to improve sleep quality. Healthcare providers need to address these symptoms individually and discuss individualized target strategies with patients and caregivers.

Our data demonstrate that most symptoms with higher prevalence are generally associated with ­greater mean burden scores, except for delusion which is somewhat frequent, but not always distressful for caregivers. Agitation and irritability are both highly prevalent and associated with significant caregiver distress, highlighting their clinical importance in care planning for healthcare professionals and caregivers. Conversely, less prevalent symptoms, such as euphoria and hallucinations, are associated with lower mean burden scores, reflecting their relatively lesser impact on caregiver distress. These findings emphasize the need to prioritize management strategies for symptoms that are both common and distressing, as they likely represent a substantial challenge in the caregiving context.

A limitation of this work is the lack of stratification by dementia type and disease stage. The included studies comprised patients across all stages of dementia and relied on a global analysis of dementia types. While this inclusive approach allowed for a broad overview of neuropsychiatric symptoms and caregiver burden, it may have limited the identification of patterns specific to particular dementia etiologies and severity levels.

In conclusion, there is a significant association between caregiver distress and neuropsychiatric symptoms in people living with dementia. The findings emphasize the importance of addressing not only the most prevalent symptoms, such as agitation, apathy, and irritability, but those that cause the most immediate and overwhelming distress to caregivers as well, including anxiety and nighttime disturbances. Tailored treatment strategies that prioritize managing these symptoms can help reduce caregiver burden, improve the quality of care, and enhance the overall well-being of both patients and caregivers. Studies should be conducted to assess the most effective approach for alleviating caregiver distress and managing the neuropsychiatric symptoms in people living with dementia.

## Data Availability

The datasets generated and/or analyzed during the current study are publicly available at PubMed, SciELO, Embase, and LILACS (https://pubmed.ncbi.nlm.nih.gov; https://www.scielo.br; https://www.embase.com; https://lilacs.bvsalud.org).
